# Supplementary material for: The association of diminished quality of life of Afghan adults’ psychosocial wellbeing, in the era of the Taliban 2.0 government
Source: PLOS Ment Health. 2025 Jan 16;2(1):e0000118. doi: 10.1371/journal.pmen.0000118 (PMC12798289; doi:10.1371/journal.pmen.0000118)
Supplement: S1 Table — (DOCX) [file pmen.0000118.s001.docx]

**S1 Table: Pairwise correlation analysis of 2022-23 Afghan psychosocial stress and quality-of-life**

|  | PSS level | Sadness | Anxiety | Withdrawal | Anger | Poor sleep | Poor eating | Nightmares |
| --- | --- | --- | --- | --- | --- | --- | --- | --- |
| Age | 0.09* | 0.04 | 0.11** | 0.11** | 0.09* | 0.09* | 0.06 | 0.06 |
| HH size | -0.09* | -0.08* | -0.09* | -0.12** | -0.03 | -0.11** | -0.02 | -0.05 |
| Sex (1 M; 2 F) | -0.02 | -0.03 | -0.05 | 0.03 | 0.00 | -0.01 | 0.02 | -0.02 |
| DQOL level | 0.54*** | 0.49*** | 0.44*** | 0.42*** | 0.36*** | 0.38*** | 0.40*** | 0.46*** |
| Enough food | 0.29*** | 0.26*** | 0.22*** | 0.19*** | 0.16*** | 0.16*** | 0.22*** | 0.26*** |
| No healthcare access | 0.32*** | 0.26*** | 0.25*** | 0.25*** | 0.19*** | 0.23*** | 0.23*** | 0.27*** |
| Infrequent contact | 0.23*** | 0.20*** | 0.18*** | 0.21*** | 0.18*** | 0.15*** | 0.20*** | 0.21*** |
| Threat of violence | 0.61*** | 0.54*** | 0.53*** | 0.45*** | 0.45*** | 0.44*** | 0.40*** | 0.50*** |
| Lost loved one | 0.33*** | 0.34*** | 0.25*** | 0.29*** | 0.20*** | 0.26*** | 0.27*** | 0.28*** |
| Comfort in religion | -0.21*** | -0.18*** | -0.20*** | -0.16*** | -0.13** | -0.16*** | -0.13** | -0.17*** |

*p<0.05, **p<0.01, ***p<0.001
