# Supplementary figures and images for: The association of diminished quality of life of Afghan adults’ psychosocial wellbeing, in the era of the Taliban 2.0 government
Source: PLOS Ment Health. 2025 Jan 16;2(1):e0000118. doi: 10.1371/journal.pmen.0000118 (PMC12798289; doi:10.1371/journal.pmen.0000118)

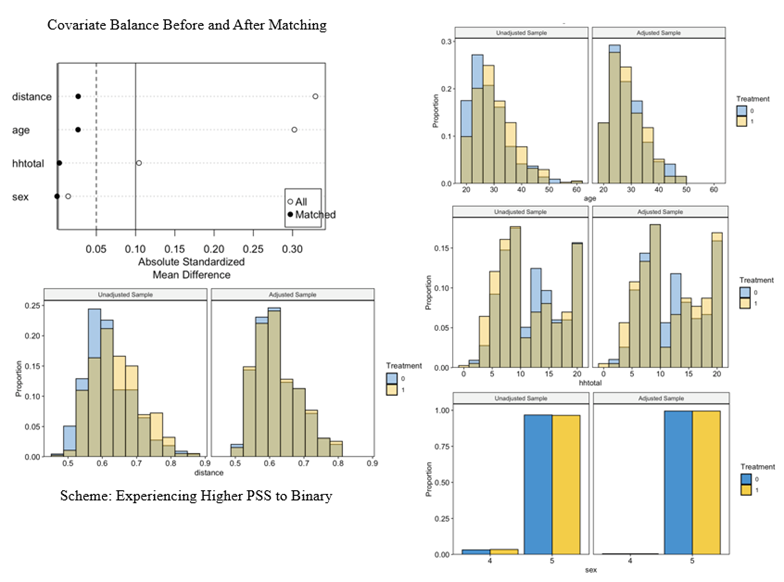

Supplement: S1 Fig — (TIF) [file pmen.0000118.s002.tif]
