# Supplementary material for: The association of diminished quality of life of Afghan adults’ psychosocial wellbeing, in the era of the Taliban 2.0 government
Source: PLOS Ment Health. 2025 Jan 16;2(1):e0000118. doi: 10.1371/journal.pmen.0000118 (PMC12798289; doi:10.1371/journal.pmen.0000118)
Supplement: S1 Text — (DOCX) [file pmen.0000118.s003.docx]

**S5 Text: Survey Questions**

| Consent- A participant's consent indicates that they have read the information provided above, that they have freely decided to participate in this research, and that they know they have not given up any of their legal rights. If you are between 18 and 64 years old, understand the statements above, and freely consent to participate in the study, click on the "yes" button to begin the survey | Yes, no (if no, end of survey) |
| --- | --- |
| Q1 Do you currently reside in the country of Afghanistan (multiple choice) | Yes; no; decline to answer |
| Q2 What is your age in years or how old are you (years) | Numerical answer years; blank |
| Q3 How many people including adults and children live in your household or house compound | Total number |
| Q4 Are you male or female? | Female (woman); male (man) |
| Q5 Are you and your family currently facing any of the following problems- not enough money to buy food; lack of healthcare, hospitals, or places to seek medical support; infrequent contact with family and friends outside of my home; threats of violence or harm; loss of family | No, not at all; yes, some days; yes, most days |
| Q6 Can you explain in your own words what your current living and working situation is like? | Open-ended |
| Q7 How is the humanitarian crisis affecting your daily life if at all? | Open-ended |
| Q8 Is there something that you do that helps you feel happy or motivated? Select all that apply - prayer, studying or reading, family and friends; television, internet or entertainment; work; love of my country | Yes, no; no response |
| Q9 Please indicate to what extent you experience the following symptoms as a direct result of the crisis in the last two years: sadness or crying; anxiety or worry; withdrawal or avoiding talking to others; anger; poor sleep or fatigue; bad eating habits (eating too much or not enough); nightmares or bad dreams | Never; infrequently; some days of the week; most days of the week (Likert) |
| Q10 What are some of your greatest challenges? | Open-ended |
| Q11 What support does the government under the Taliban provide to citizens? | Open-ended |
| Q12 Do you find spiritual solace or comfort from your religion? | Yes, a lot of comfort; yes, I get some comfort; no, none at all; no response |
| Q13 How are the roles of women and men different? | Open-ended |
| Q14 Do you have any additional information that you would like to share? | Open-ended |
