# Supplementary material for: The association of diminished quality of life of Afghan adults’ psychosocial wellbeing, in the era of the Taliban 2.0 government
Source: PLOS Ment Health. 2025 Jan 16;2(1):e0000118. doi: 10.1371/journal.pmen.0000118 (PMC12798289; doi:10.1371/journal.pmen.0000118)
Supplement: Attachment — Submitted filename: Reviewer feedback.docx [file pmen.0000118.s004.docx]

Editor Feedback

1. Please include a complete copy of PLOS’ questionnaire on inclusivity in global research in your revised manuscript. Our policy for research in this area aims to improve transparency in the reporting of research performed outside of researchers’ own country or community. The policy applies to researchers who have travelled to a different country to conduct research, research with Indigenous populations or their lands, and research on cultural artefacts. The questionnaire can also be requested at the journal’s discretion for any other submissions, even if these conditions are not met.  Please find more information on the policy and a link to download a blank copy of the questionnaire here: https://journals.plos.org/plosmentalhealth/s/best-practices-in-research-reporting. Please upload a completed version of your questionnaire as Supporting Information when you resubmit your manuscript.

Response: completed

2. Please amend your detailed Financial Disclosure statement. This is published with the article. It must therefore be completed in full sentences and contain the exact wording you wish to be published. **Please only choose the relevant sentences from below** 1. Please clarify all sources of funding (financial or material support) for your study. List the grants (with grant number) or organizations (with url) that supported your study, including funding received from your institution.  2. State the initials, alongside each funding source, of each author to receive each grant. 3. State what role the funders took in the study. If the funders had no role in your study, please state: “The funders had no role in study design, data collection and analysis, decision to publish, or preparation of the manuscript.” 4. If any authors received a salary from any of your funders, please state which authors and which funders. If you did not receive any funding for this study, please simply state: “The authors received no specific funding for this work.”

 Response: completed

“NK and HS received the 2023 Hobson Wildenthal Honors College Undergraduate Research Apprenticeship Program (URAP) grant to serve as co-researchers on this project, from the University of Texas at Dallas (<https://honors.utdallas.edu/research/undergraduate-research-apprenticeship-program/>).

The funders had no role in study design, data collection and analysis, decision to publish, or preparation of the manuscript. No other authors received specific funding for this work.”

3. We note that you have indicated that there are restrictions to data sharing for this study. For studies involving human research participant data or other sensitive data, we encourage authors to share de-identified or anonymized data. However, when data cannot be publicly shared for ethical reasons, we allow authors to make their data sets available upon request. For information on unacceptable data access restrictions, please see http://journals.plos.org/plosone/s/data-availability#loc-unacceptable-data-access-restrictions.  Before we proceed with your manuscript, please address the following prompts:  a) If there are ethical or legal restrictions on sharing a de-identified data set, please explain them in detail (e.g., data contain potentially identifying or sensitive patient information, data are owned by a third-party organization, etc.) and who has imposed them (e.g., a Research Ethics Committee or Institutional Review Board, etc.). Please also provide contact information for a data access committee, ethics committee, or other institutional body to which data requests may be sent.  b) If there are no restrictions, please upload the minimal anonymized data set necessary to replicate your study findings to a stable, public repository and provide us with the relevant URLs, DOIs, or accession numbers. Please see http://www.bmj.com/content/340/bmj.c181.long for guidelines on how to de-identify and prepare clinical data for publication. For a list of recommended repositories, please see https://journals.plos.org/plosone/s/recommended-repositories. You also have the option of uploading the data as Supporting Information files, but we would recommend depositing data directly to a data repository if possible.  Please update your Data Availability statement in the submission form accordingly.

Response: We have consulted this and this is the statement approved:

“There are ethical restrictions on sharing a de-identified data set, as data contain potentially identifying or sensitive participant information, as mandated by the University of Texas Institutional Review Board and Office of Human Subjects Protections (OHSP) (contact information [research@utdallas.edu](mailto:research@utdallas.edu)). Additionally, while the study was anonymous, the participants of this study were informed that their data would not be shared publicly, thus they did not give written consent. Due to the sensitive nature of the research, supporting data is not available”

4. Please provide separate figure files in .tif or .eps format.

 Response: completed

For more information about figure files please see our guidelines:

https://journals.plos.org/mentalhealth/s/figures

https://journals.plos.org/mentalhealth/s/figures#loc-file-requirements

5. Tables should not be uploaded as individual files. Please remove these files and include the Tables in your manuscript file as editable, cell-based objects. For more information about how to format tables, see our guidelines:

 Response: completed

https://journals.plos.org/mentalhealth/s/tables

6. We have noticed that you have uploaded Supporting Information files, but you have not included a list of legends. Please add a full list of legends for your Supporting Information files after the references list.

  Response: completed

7. We notice that your supplementary figures are uploaded with the file type 'Figure'. Please amend the file type to 'Supporting Information'. Please ensure that each Supporting Information file has a legend listed in the manuscript after the references list.

 Response: completed

**Reviewer 1:**

Keywords: The term "new Taliban" may be misleading; it might be more appropriate to refer to this as the "new Taliban era." While I understand the intention behind "new," this phrasing could lead to confusion.

Response: Thank you dearly for pointing this out. We will use it much less and instead refer to the name of the government assigned by the Taliban after the 2021 takeover. We determined to call it Taliban- Islamic Emirate of Afghanistan or Taliban 2.0

- Results: The last sentence regarding qualitative data would be better placed in the Methods section rather than in Results.

Response: We have done this. We changed:

“Against this backdrop, our study seeks to offer a more contemporary analysis of DQOL experiences and psychosocial stress (PSS), years into the transition of power. We present 2023 digital survey data collected from 873 respondents living through the humanitarian crisis in their homeland. In the next section, we present the methodology, including the theoretical framework, survey design, data collection, and analysis. The rest of this article is presented as follows: the Results section presents the statistical findings (correlation analysis, t-test analysis, multivariate regression analysis, logistic (odds-ratio), and matching modelling) related to the research questions. The Discussion section explores the quantitative findings in more detail related to relevant scholarship as well as qualitative statement made by respondents- the purpose of which is to triangulate the findings and to help provide a deeper insight to the causal mechanisms [33–35]. We conclude the paper by presenting research implications and limitations, along with proposed policy recommendations.

# Material and methods

## Purpose of this Study

This study examines the following questions:

1. To what extent do Afghan adults (ages 18-65), living in the era of the Taliban 2.0, self-report experiencing ongoing DQOL (measured by disaggregated common hardships and aggregated levels)?
2. To what extent does DQOL (aggregated and disaggregates experiences of poorer access to: a) food and b) healthcare, c) infrequent contact with loved ones, d) lost family, e) threat of violence) affect their psychosocial stress (measured by the Perceived Risk Scale; aggregated and disaggregated by gender, age range, household conditions, and religiosity)?

Based on the commonalities within relevant literature, we test the following hypotheses:

H1: Afghan adults experiencing higher rates of physical hardships related to behavioral & health system factors (food insecurity, violence, & limited healthcare access) are correlated to higher PSS (individual stressors and aggregate)

H2: Afghan adults who experience high rates of psychosocial circumstances (loss of loved ones, limited social networks) are correlated to higher PSS

H3: Those who experience higher rates/frequency of DQOL factors and aggregated DQOL exhibit higher rates of PSS”

Introduction:

- NATO should be spelled out upon first use.

Response: This is done:

“after nearly 20 years of the US- North Atlantic Treaty Organization (NATO) involvement in the ‘War on Terror,’ which the Taliban historically has referred to as an occupation”

- There are several sentences that begin with numbers in both the abstract and the body. For improved readability, I suggest writing out the numbers or using a word before them.

Response: Thank you. We hired an editor to go through this to make that this is done where best recommended.

- The phrase "the purpose of this study" would fit better as a subsection of the Methods section. It’s helpful to have a general statement of purpose at the end of the introduction, but detailed information is best kept in the Methods section.

Response: This is done. See above.

Methods:

- In the participants section (page 7), some results are mentioned. It would be more appropriate to focus solely on the methods and avoid including results in this part.

Response: Wonderful suggestion. This is completed. This was moved under the start of the Results.

“**Unadjusted quantitative analysis**

Of the 843 survey participants who met all criteria to be included in this analysis, 794 (94.2%) self-report being men, 28 (3.3%) women, and 21 (2.5%) do not disclose. 53.7% are aged 18-29; 34.2% are 30-39; 9.7% are 40-49; and 2.4% are 50-65. The average household size is 11.7 people (95%CI 11.3-12.0).”

Reviewer #2: Dear authors,

This is an important contribution to the literature given the paucity of data on Afghanistan. I have a few comments that I would like your clarification on.

1: I'm curious about the absence of a reflexivity statement. Given the sensitive sociopolitical context of Afghan adults' quality of life under the Taliban regime, including such a statement would provide transparency regarding the authors' relationship to the context. An awareness of researchers' positionality can better inform readers about potential influences on data collection and analysis.
Response: Thank you- we have added this:

**Researcher characteristics and reflexivity**

The research team comprised academic researchers, university student researcher assistants, and nonprofit practitioners from the United States, Afghanistan, Russia, and China. Table 2 describes the diversity and strength of all the authors informing the credibility of the results. All authors have social science academic backgrounds. Two co-authors are Afghan, and led in the translation, validation, and pilot testing, and data analysis including the qualitative analysis of the survey including open-ended questions. One researcher is directly impacted by the conditions in Afghanistan, including facing violence, limited work, and food insecurity. The second is a student in the US with family and colleagues still residing in Afghanistan. The US-based academics supported the design in English, data collection and analysis. Most have experience conducting rigor mixed-method, survey research on MHPSS and DQOL in low-income settings affected by extreme poverty, civil conflict, and humanitarian hardships. All have some academic training and work experience in Western institutions. We created a plan to avoid a neocolonial research dynamic on this project and meet to discuss our progress and any concerns. Our diverse cultural backgrounds help provide a robust lens to this research, but we prioritize the expertise, analysis, and feedback of our Afghan co-authors in this entire research process and analysis.

**Table 2** Characteristics of team members

| JD | American PhD in policy and global health policy; professor in American university; female; Caucasian; 20 years of international development work experience in Africa; Latin America; Asia in human rights and mixed-method field research; focusing on MPHSS and DQOL needs during civil crises; supported development project support and research on Afghanistan for three years |
| --- | --- |
| AS | Russian PhD in political science; Caucasian; male; advanced statistical analysis skills; professor in American university researching questions of politics using text analysis; machine learning; and causal inference; research projects focus on mass protest; cybersecurity; and political control in autocracies; moderate familiarity with Afghan politics |
| SS | Afghan PhD student at US university; male; former Afghan policy advisor in Kabul before 2021 US withdrawal; family still in Afghanistan and US; fluent in major Afghan languages; research focus on political science; well-connected on social media with political channels; expertise in working with international institutions in development and policy projects in Afghanistan |
| AM | American nonprofit practitioner; 3 years’ experience working in Africa; little experience with Afghan issues; led mixed method field research in low-income countries; master’s degree in human rights and nonprofit; white; female; experience in conducting research on MHPSS and DQOL in conflict |
| HP | Afghan; male; BA from Afghan university; former translator for US nonprofits; Pashto; living in Afghanistan; fluent in multiple Afghan languages; family impacted by humanitarian crisis in country; expertise on conducting research in country and online in collaboration with national and international co-researchers |
| HS | Indian American; female; pre-medical university and biology student in USA; focus of research is in healthcare policy and global health equity; volunteering to support nonprofits focused on stopping gender-based violence and humanitarian crises including in Uganda, Rwanda, and Afghanistan |
| MS | Chinese; female; PhD in political science with background in machine learning, deep learning, time-series, and other quantitative statistical models to evaluate the competitive trade relations between the US and China; support in co-authoring research related to DQOL, health crises, and conflict |
| NK | Indian American; female; university student in political sciences; research interests in improving global health inequities in USA and low-income countries in MENA and south-east Asia; research experience on humanitarian crisis in Afghanistan |

2: Could you provide more information about the questionnaire development process? Specifically:
a) What sources were used to develop the questionnaire?
b) How was it piloted?
c) Were internal and external validity assessed?
d) Did the scales undergo cultural adaptation?

Response: Thank you, good advice. We have added the entire survey in the Appendices. Additionally, we detailed this more:

## “

## Data collection process and study population

A robust literature review of 231 top-cited studies on Afghan QoL was performed on Google Scholar and PubMed (using search terms including *Afghanistan, humanitarian crisis, war, mental health, psychosocial wellbeing, PSS, quality of life, study)* by the PI and CIs to identify key QOL variables for the analysis, as well as an in-depth contextual understanding of the political, economic, and social history of Afghanistan. The survey closed-ended and open-ended questions were translated and piloted with lead facilitation by our Afghan co-authors before launching data collection, including assessing for contextual and cultural relevance. Approximately a dozen adult Afghans living in the host-country and abroad voluntarily participated in the pilot-test, with all feedback incorporated to minimize threats to validity, including feedback to limit any demographic questions pertaining to ethnicity or direct political affiliations as they were often perceived as triggering. Pilot tested data was excluded from the analysis.

The survey was translated and presented in Pashto, Dari, and English (translation verified by Afghan translator and pilot-testing process). As part of the ethical approval process, we selected to present the study design and survey electronically with the Ministry of Higher Education and the Ministry of Public Health (MoPH) of the 2022 Afghan Government to ensure adherence to ethical protocols to responsible practices and safeguard of participants' privacy and safety given the political climate and strict media regulations. No formal response was received by the Afghan ministries. Data analysis indicates that numerous participants who took the survey worked for the Afghan government or military and/or held pro-Islamic Emirate of Afghanistan affiliations. Participation eligibility criteria included fluency in at least one language, being between the ages of 18-65, residing within Afghanistan at the time, and completing the IRB consent process. Data collection occurred from July to December of 2023, using a snowball sampling design of sharing the recruitment script in all three languages over Facebook (in 2021 approximately 68% of Afghans own mobile phones/iPhones and millions use social media, with subscription increases in 2022 [36, 37]). The survey was then shared on over 50 publicly open Facebook pages related to Afghan news, regional/community pages, university sites, education boards, governmental sites, and human rights pages. Participants could move between questions, and decline to answer all open-ended questions, and select ‘decline to answer’ or ‘n/a’.

## Instrumentalization

We designed and launched a digital survey with both close-ended/multiple choice questions related to PSS, demographics, and DQOL factors, and additional open-ended questions where participants could elaborate if they chose, or they could leave it blank. No interviewers were used, and the open-ended questions were based on similar DQOL questions from our research, as well as adapted from USAID-DHS survey applied in similar Afghan studies [38]. The digital survey includes multiple choice questions measuring participants’ self-reported demographics (age, gender, resident within country status, household size), QoL factors, and PSS levels (translated in all three languages) (see Appendix 4 for all questions). Survey questions related to MHPSS were based on Foa (2001) psychosocial stress scale (PSS), often used for participant self-reporting stress, [39–41]. This instrument was also adopted, modified, and tested in various studies including in post-war, crisis in low-income nations including Afghanistan, Ukraine, and Liberia [39–45].

PSS scales are regularly used to assess psychosocial stress self-reported by participants experiencing increased trauma events [26, 28, 39, 40]. These scales capture individual stressors like anxiety, extreme anger, or withdrawal, using Likert scaling, which can be assessed both disaggregated and aggregated, testing for Cronbach’s alpha. Additionally, QOL factors are also assessed using a Likert scale, assessing both individual and aggregated levels (see Table 1).

**Table 1** The reliability test of study variables & scales

| **Variable** | **Description** | **Cronbach’s α** | **Pearson R coefficient-overall score** |
| --- | --- | --- | --- |
| **PSS Level** | **Total Likert score of all stressors (0 none to 21 high)** | **0.89** |  |
| Sadness | Person’s weekly experiences (0 never, 1 some days, 2 most days/week) | 0.88 | 0.77*** |
| Anxiety/worry |  | 0.86 | 0.82*** |
| Withdrawal/ avoiding people |  | 0.87 | 0.77*** |
| Anger |  | 0.87 | 0.78*** |
| Poor sleeping habits |  | 0.86 | 0.81*** |
| Poor eating habits (too much or too little) |  | 0.88 | 0.74*** |
| Nightmares |  | 0.88 | 0.74*** |
| **QoL hardship level** | **Aggregate total of factors (0 none-10 high)** | **0.70** |  |
| Not able to buy enough food | (0 never, 1 some days, 2 most days/week) | 0.65 | 0.69*** |
| Not able to access healthcare services or medicine |  | 0.64 | 0.70*** |
| Infrequent contact with friends and family outside of HH |  | 0.66 | 0.67*** |
| Face threat of harm or violence |  | 0.65 | 0.71*** |
| Bereavement for lost loved ones |  | 0.67 | 0.62*** |

Note: ^***^*p* < 0.001. Qol= quality of life factor. PSS= psychosocial stress symptom.

## Participants

Of the 1798 people who opened the survey, 891 respondents consented to take part and provided self-reported information. 22 were excluded as they stated that they resided outside of Afghanistan, along with 26 respondents who did not disclose their location. This study only considers within-country populations at the time of the study. An initial review of open-ended question responses indicates a balanced distribution of participants who worked for or held political affinities for the deposed government and the American forces, compared to those who work for or are in favor of the Taliban 2.0 government/ Islamic Emirate of Afghanistan. Data on specific location, language, and ethnicity were not collected for ethical reasons presented by Afghan consultants, who urged that such questions would make many respondents feel uncomfortable disclosing and/or fearful of the study’s intent in current political climate and the historical conflict between specific ethnic groups. Future studies may consider including additional demographic variables. This analysis purposefully examines Afghans as a country of citizens under a new era, often facing similar hardships irrespective of their political or ethnic background.

## Data analysis

For this mixed-method study, we conducted a statistical analysis using Stata, including descriptive statistics, pairwise correlation analysis, and multivariate regression modelling of all quantitative survey questions. Based on the snowball method applied, the survey is not randomized, limiting data generalizability. Thus, we further adjust for covariate imbalances through OLS and Matching to further test casual patterns asserted in the unadjusted analysis. The integrity of any observational study hinges on the premise that the treatment and control groups are comparable on all observed covariates [46–48]. Recognizing some initial imbalances in our dataset, we embarked on a two-pronged approach to mitigate these discrepancies.

First, we adjusted for covariates within our regression models. This process is pivotal for controlling confounding variables that could otherwise distort the true effect of the treatment on the outcome. The adjusted model showed improvements in covariate balance, indicating a reduction in potential biases that could affect the results. Second, we employed a matching strategy to create a subsample of treated and control observations that were more alike, mirroring the covariate distribution in the treatment group across the control group, hence reducing selection bias. Matching involves pairing individuals from the treatment group with those in the control group based on similarity across a set of covariates, thereby reducing baseline differences.

Thirdly, we transitioned from traditional regression coefficients to Marginal Effects, focusing on the Average Treatment Effects (ATE) and their Standard Errors (SE). This transition allows us to convey the impact of the independent variables on the probability of experiencing increased PSS in a more intuitive manner. Marginal Effects were calculated using the ‘mfx’ package in R, which simplifies the process of converting logistic regression coefficients into average marginal effects. This method involves estimating how a slight change in each predictor variable, holding all other variables constant, would affect the probability of the outcome variable.

Lastly, we conduct a qualitative analysis of participant responses to open-ended survey questions to triangulate the quantitative findings, and to explore in depth the meaning behind their life experiences, their struggles, and needs living in Afghanistan after the withdrawal of American forces [33, 35, 49, 50]. All data analysis, discussion, and conclusions were supported by all co-investigators, with our Afghan colleagues’ interpretations, translations, and expertise prioritized. The qualitative findings are elaborated upon in the Discussion.”

3: Regarding the qualitative surveys:

a) How were they conducted?
b) Who conducted them?
c) Are the interviewers' contributions acknowledged?
d) What tools were used for qualitative data analysis?

No interviews were conducted. This was a digital survey. But we used Excel for the qualitative data analysis. Modified section:

“Lastly, we conduct a qualitative analysis of participant responses to open-ended survey questions to triangulate the quantitative findings, and to explore in depth the meaning behind their life experiences, their struggles, and needs living in Afghanistan after the withdrawal of American forces [33, 35, 49, 50]. The qualitative data analysis was conducted using Excel, in which for data cleaning, notes taking, capturing emerging trends, including coding and tracking these themes. The research team assessed in weekly sessions to analyze and explore these themes together. All data analysis, discussion, and conclusions were supported by all co-investigators, with our Afghan colleagues’ interpretations, translations, and expertise prioritized. The qualitative findings are elaborated upon in the Discussion.”

4: While you employed matching techniques to control for confounding variables, a more detailed justification for the selection of specific control variables is essential. For instance, controlling for region (urban vs. rural), education level, or political affiliations could add depth, as these factors might directly impact stress levels under the current political regime.

Response: Thank you: we have added this as an appendix and noted for more detail:

Response by our statistician: While you employed matching techniques to control for confounding variables, a more detailed justification for the selection of specific control variables is essential. For instance, controlling for region (urban vs. rural), education level, or political affiliations could add depth, as these factors might directly impact stress levels under the current political regime.

Thank you for this suggestion. While the inclusion of additional variables could enhance the depth of analysis, the constraints of the current environment necessitated a cautious approach. Our non-ideal methodological choices were mainly influenced by two factors:

Ethical and Safety Considerations in Data Collection:

In the current Afghan context, certain questions that might be benign elsewhere can pose significant risks to respondents. As we state in the paper (245), inquiring about specific locations, languages, or ethnicities could lead to discomfort or fear due to historical conflicts and the prevailing political climate. Academic literature supports this caution. For instance, Blair, Imai, and Lyall (2014) discuss the challenges of collecting sensitive data in conflict zones, emphasizing the need to protect respondent anonymity to ensure safety. Their other work (2013) highlights the importance of survey experiments to control biases and the risks such variables may introduce in observational data, as in our case. Using indirect questioning techniques can mitigate risks in sensitive environments.

Optimizing Questionnaire Length to Enhance Response Rates:

We aimed to balance the depth of information with respondent engagement by limiting the questionnaire length. Research indicates that in environments like Afghanistan under the Taliban, shorter surveys tend to achieve higher response rates. For example, Galesic and Bosnjak (2009), Fan and Yan (2010), and Holtom et al. (2022) found that longer questionnaires significantly decrease participation rates in similar contexts.

We absolutely agree that adjusting for additional regional-level and individual-level factors would improve the transparency of our results and might provide further insights into the mechanisms we study in this paper. As we note on page 6, "future studies may consider including additional demographic variables." Including an "education" variable, in particular, could have offered especially valuable insights for our study, and we regret not incorporating it into the questionnaire perhaps at the price of a lower response rate.

5: The results mention qualitative data "triangulation," but the integration appears limited, with qualitative findings seeming secondary to quantitative outcomes. Please consider expanding the qualitative results section or using quotes to illustrate quantitative trends. This would add richness and narrative depth, particularly given the study's mixed-methods nature.

Response: Yes, this is a good point that we debated regularly about. We have decided to do a series of papers after focusing on the statistical trends, and each additional paper focusing using a narrative analysis approach to explore each trend. We added to our Limitations section: “Lastly, our paper utilizes the qualitative data for "triangulation," with qualitative findings secondary to quantitative outcomes. This paper seeks to first establish statistical trends, while mainly using quotes to illustrate quantitative trends. Future research will provide a more in-depth analysis of the qualitative data, using a narrative approach.”


Good luck!!
